# Association of the P441L KCNQ1 variant with severity of long QT syndrome and risk of cardiac events

**DOI:** 10.3389/fcvm.2022.922335

**Published:** 2022-10-31

**Authors:** Haoyang Lu, Wen Ding, Hui Xiao, Manyu Dai, Yangcheng Xue, Zhuoran Jia, Jie Guo, Mengzuo Wu, Bing Shen, Ren Zhao

**Affiliations:** ^1^Department of Cardiovascular Medicine, The First Affiliated Hospital of Anhui Medical University, Hefei, China; ^2^School of Basic Medical Sciences, Anhui Medical University, Hefei, China

**Keywords:** long QT syndrome, *KCNQ1*, I_*Ks*_, channel trafficking, C-terminus mutation

## Abstract

Dysfunction of potassium voltage-gated channel subfamily Q member 1 (KCNQ1) is a primary cause of long QT syndrome type 1 (LQT1). Here, we report a missense mutation P441L in KCNQ1 C-terminus of a 37-year-old woman with severe LQT1 phenotype. Variant P441L transporting to the plasma membrane and interacting with KCNE1 were both markedly decreased, leading to potassium efflux disorder and eventually LQT1. Mutations between the C-terminal helix A and helix B of KCNQ1 have linked with low cardiac event risk, however, we firstly find variant P441L causing a severe LQT1 phenotype with a high risk of cardiac events.

## Introduction

Long QT syndrome (LQTS) is a hereditary heart disease caused by gene variants that lead to abnormal coding of channel proteins that cause prolonged myocardial repolarization time (1, 2). A prolonged QT interval on a surface 12-lead electrocardiogram (ECG) characterizes LQTS, often being in the form of torsade de pointes, which may lead to sudden cardiac death (3, 4). Type 1 LQTS (LQT1) is the most common type of congenital LQTS, accounting for approximately 45% of cases (5). Potassium voltage-gated channel subfamily Q member 1 (KCNQ1), encoded by the *KCNQ1* gene, is an α subunit of a voltage-gated potassium (K^+^) channel that forms a channel pore mediating K^+^ efflux and cardiomyocyte repolarization in the plateau phase of cardiac action potentials. The KCNQ1 protein associates with the regulatory β subunit of potassium voltage-gated channel subfamily E regulatory subunit 1 (KCNE1) to mediate the slow delayed rectifier cardiac current (I_*Ks*_) (6, 7). Dysfunction of KCNQ1 is a primary cause of LQT1 (8).

The C-terminal domain of the *KCNQ1* gene contains four helical regions A–D, which are responsible for channel gating, assembly, and transport (9–11). Variants located at different positions of the gene are associated with varying degrees of severity in clinical symptoms. Therefore, it is particularly important to explore the associations between *KCNQ1* genotype and clinical phenotype. In addition, identification of LQT1 pathogenesis may provide a reliable theoretical basis for treatment. In the present study, we identified a C-terminal *KCNQ1* mutation (*P441L*) in a woman 37 years of age who was diagnosed as having LQTS with a severe clinical phenotype.

## Materials and methods

### Ethical considerations

This study was approved by the Institutional Review Committee of the First Affiliated Hospital of Anhui Medical University (PJ2021-14-34). The patient provided written informed consent before peripheral blood was collected. This study complies with the principles set forth in the Declaration of Helsinki.

### Electrocardiogram analysis

A surface 12-lead ECG was synchronously traced with a Nalong-12PL electrocardiograph at a paper speed of 25 mm/s. The QT interval was determined from the start of the QRS wave to the end of the T wave.

### *KCNQ1* genomic analysis

Genomic DNA was isolated from the patient’s peripheral blood leukocytes. Polymerase chain reaction and Sanger sequencing were performed. The forward primer was 5′-ATCTGCTTCCTGCTGTCCTG-3′, and the reverse primer was 5′-TGGCTTGCTCGGGCTCCCAA-3′. The sequencing reaction was performed using an ABI 3730 DNA analyzer (Thermo Fisher Scientific, Waltham, MA). The obtained chromatograms were analyzed using Variant Reporter, version 1.1 (Applied Biosystems, Thermo Fisher Scientific).

### Plasmid construction, point mutation, and transfection

The full-length cDNAs for *KCNQ1* (GenBank NM_000218) and *KCNE1* (GenBank NM_000219) were obtained using a high-fidelity polymerase chain reaction DNA polymerase (P505-d1, Novizan, China). The *KCNQ1* cDNA was cloned into a pEGFP-N1 vector, and the KCNE1 cDNA was cloned into a pmCherry-N1 vector. We used a KCNQ1-EGFP plasmid as a template and a point mutation kit (C214-01, Novizan, China) to mutate the 1322nd base in the coding sequence region of the *KCNQ1* gene from C to T, and the 441st amino acid from proline (P) to leucine (L). After the point mutations were completed, the sequences were transfected into human embryonic kidney (HEK293) cells.

### Electrophysiology

We transferred glass coverslips with the transfected HEK293 cells to 500 μL of a solution containing (in mM) 150 NaCl, 5 KCl, 1 MgCl_2_, 2.5 CaCl_2_, 10 HEPES, and 10 glucose (pH 7.4, adjusted with NaOH). Glass pipette electrodes were pulled from borosilicate glass (1B150F-4, World Precision Instruments, USA) with a glass electrode puller (PC-100, Narishige, Japan), to a resistance of 3–5 MΩ. The intracellular solution used in the pipette contained (in mmol/L) 153 KCl, 1 MgCl_2_, 10 HEPES, 5 EGTA, and 4 Na_2_ATP (pH 7.2, adjusted with KOH). An EPC-9 amplifier controlled by PatchMaster software (HEKA, Lambrecht, Germany) was used to measure the whole-cell current of EGFP/mCherry–positive cells. The holding voltage was −80 mV.

### Confocal microscopy

After transfection, HEK293 cells were incubated with endoplasmic reticulum (ER)-Tracker Red (C1041, Beyotime, China) at 37°C for 30 min and then fixed with 4% paraformaldehyde. Following three washouts with phosphate-buffered saline, 4′,6-diamidino-2-phenylindole (DAPI) (C1005, Beyotime, China) was used to stain cell nuclei. HEK293 cells transiently transfected with KCNQ1-EGFP/KCNE1-mCherry and P441L-EGFP/KCNE1-mCherry were also stained with DAPI. An argon laser was used to excite EGFP and mCherry, and an oil microscope objective was used to image the cells. An LSM 880 (Leica, Germany) laser scanning confocal microscope was used to obtain merged images by superimposing images from different channels.

### Co-immunoprecipitation and western blotting

Non-idet P-40 lysis buffer was added to lyse the cells. Protein A/G Magnetic Beads (HY-K0202, MedChemExpress, USA) and KCNQ1 antibody (sc-365186, Santa Cruz, USA) were incubated at 4°C for 2 h to allow for the formation of magnetic bead–antibody complexes. The supernatant was incubated with the magnetic bead–antibody complexes on a shaker at 4°C for 3 h. After a washout, the obtained Co-IP product was resuspended in 100 μL of loading buffer and electrophoresed using 8% gels by sodium lauryl sulfate–polyacrylamide gel electrophoresis. The separated proteins were transferred to polyvinylidene fluoride membranes. The membrane was incubated with anti-KCNQ1 and anti-KCNE1 antibodies (DF6310, affinity, China) overnight at 4°C. After being rinsed, membranes were incubated with goat anti-rabbit and goat anti-mouse IgG horseradish peroxidase–conjugated secondary antibodies (1:5000) for 2 h at room temperature. Enhanced chemiluminescence substrate was used to detect the labeled secondary antibodies, and protein bands were quantified by densitometry using ImageJ software (National Institutes of Health, Bethesda, Maryland, USA).

### Statistical analysis

Data are presented as means ± SEM. Statistical analyses were performed with GraphPad Prism, version 6 (GraphPad Software Inc.), and R (The R Foundation). We conducted two-tailed Mann–Whitney tests or two-way analyses of variance followed by Games–Howell *post-hoc* tests. Statistical significance was set at *P* < 0.05.

## Results

### Clinical characteristics of the patient

**FIGURE 1:**
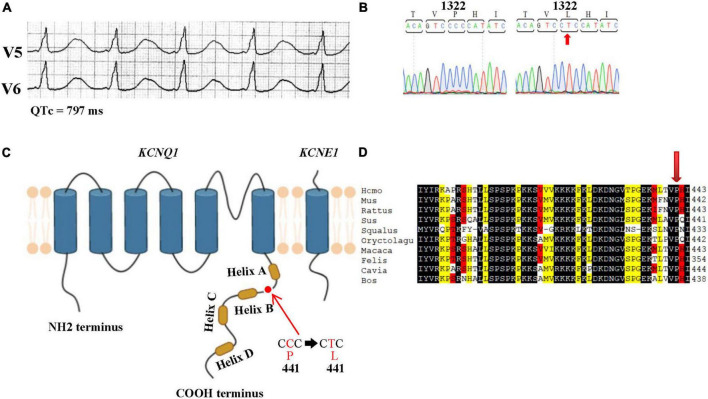
Characterization of long QT syndrome type 1 in a patient with the P441L variant of *KCNQ1* (potassium voltage-gated channel, subfamily Q member 1). **(A)** Patient electrocardiogram showing the corrected QT interval (QTc) interval is prolonged to 797 ms. **(B)** Sanger sequencing data showing the novel *KCNQ1* mutation of C1322T in the P441L variant. Sequence analysis of genomic *KCNQ1* obtained from the blood of the patient reveals a heterozygote missense mutation (C→T) at position 1322 of the *KCNQ1* coding region (C1322T). **(C)** Schematic representation of KCNQ1 protein with the P441L variant; proline is replaced by leucine at position 441 of the cytoplasmic C-terminal domain (P441L). Created with BioRender.com. **(D)** Partial amino acid sequences of different species KCNQ1 homologs aligned at the position corresponding to tyrosine 441 of human KCNQ1 (arrow). Number, the position of the last amino acid of each partial sequence. The conserved motif is outlined in black.

The patient was admitted to the hospital with repeated syncope for 2 days without obvious inducement. ECG showed sinus rhythm with a heart rate of 56 beats per min (bmp) and a QTc interval of 797 ms (Figure 1A). She had not previously used QT-prolonging drugs. During hospitalization, the patient had three additional episodes of syncope accompanied by loss of consciousness, stiff limbs, and convulsions. ECG monitoring showed torsade de pointes ventricular tachycardia, which was terminated after electrical cardioversion, and a temporary pacemaker was implanted. The patient had no discomfort, fever, dyspnea, or chest pain. No electrolyte abnormalities were detected after admission. Physical examination and transthoracic echocardiography findings were within reference ranges. The diagnosis of LQT1 was performed by DNA sequence identifying an allelic missense mutation C1322T (Figure 1B), Among mammalian homologs, the species of the amino acid corresponding to P441 of human KCNQ1 subunit are conserved, but not conserved in the KCNQ family (KCNQ2-5) (Figures 1C,D and Supplementary Figure 1).

### Effects of variant P441L on KCNQ1 channel protein trafficking in the absence or presence of KCNE1 and the interaction between P441L and KCNE1

**FIGURE 2:**
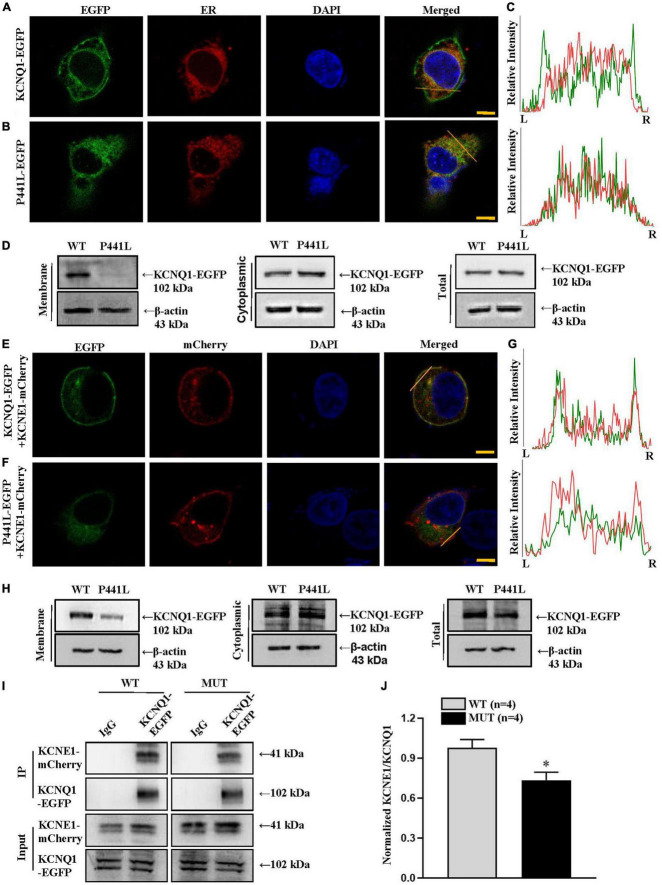
KCNQ1 P441L variant protein trafficking in the absence or presence of KCNE1 (potassium voltage-gated channel subfamily E regulatory subunit 1), and the interaction between the KCNQ1 P441L variant and KCNE1 proteins. **(A–C)** Representative confocal microscopy images and line plots showing the cellular distribution of wild-type and P441L variant KCNQ1 proteins in HEK293 cells. Scale bar, 10 μm. KCNQ1 was tagged with EGFP at the N-terminus and thus is shown as green fluorescence. Red fluorescence indicates the endoplasmic reticulum (ER); blue fluorescence, 4′,6-diamidino-2-phenylindole (DAPI), indicates cell nuclei. Line plots in panel **(C)** represent profiles plotted from left to right (L–R) to display the relative red and green fluorescence intensities obtained at the orange lines shown in the merged images. **(D)** Representative Western blot images showing fractionation assay results indicating that compared with the wild-type KCNQ1-EGFP (WT) protein level, the P441L-EGFP (P441L) protein level is significantly lower in the cell membrane but significantly increased in the cytoplasm. **(E–G)** Representative confocal microscopy images and line plots showing the cellular distribution of WT and P441L variant KCNQ1 proteins in HEK293 cells in the presence of KCNE1. Scale bar, 10 μm. HEK293 cells were transfected with KCNQ1-EGFP/KCNE1-mCherry **(E)**, or P441L-EGFP/KCNE1-mCherry **(F)**. Green fluorescence indicates KCNQ1; red fluorescence indicates KCNE1; and blue fluorescence, 4′,6-diamidino-2-phenylindole (DAPI), indicates cell nuclei. Line plots in panel **(G)** represent profiles plotted from left to right (L-R) to display the relative red and green fluorescence intensities obtained at the orange lines shown in the merged images. **(H)** Representative Western blot images showing fractionation assay results indicating that only a small amount of expressed P441L-EGFP protein is detected on the cell membrane when P441L-EGFP/KCNE1-mCherry are co-transfected into HEK293 cells. **(I)** Representative co-immunoprecipitation (IP) images showing KCNQ1 and KCNE1 proteins in lysates of HEK293 cells. In KCNQ1-EGFP/KCNE1-mCherry (WT) or P441L-EGFP/KCNE1-mCherry (MUT) expressed in HEK293 cells, the products of the IP showing KCNE1 pulled down by WT KCNQ1 or by the P441L variant. **(J)** Summary data showing the ratio of KCNE1 to WT KCNQ1 or the P441L variant pulled down IP products. WT represents KCNE1/KCNQ1; MUT, KCNE1/P441L variant. Data are shown as the mean ± SEM, *n* = 4, *P < 0.05, WT vs. MUT.

Previous studies have determined that EGFP fused to the KCNQ1 C-terminal end will not affect channel transport function (12). Our data showed that KCNQ1-EGFP (green fluorescence) was located on the cell membrane, but the P441L KCNQ1 variant was not and observed in the cytoplasm (Figures 2A–C). To confirm this finding, we extract cell membrane proteins and cytoplasmic proteins, respectively. The results indicated that compared with the level of the KCNQ1-EGFP protein, the protein level of P441L-EGFP was significantly decreased on the cell membrane but increased in the cytoplasm (Figure 2D). The findings indicated that the channel subunit with variant P441L associated with LQTS in the patient was not transported to the cell membrane but remained in the ER.

In cardiomyocytes, the slowly activating delayed rectifier K + current (I_*Ks*_) channel complex is formed by the co-assembly of KCNQ1, KCNE1, and several auxiliary regulatory molecules (6, 13). We examined that in cells with P441L-EGFP/KCNE1-mCherry (Figures 2E–G), red fluorescence indicative of KCNE1-mCherry was found predominantly in the cell membrane, but only weak green fluorescence indicative of P441L-EGFP was observed in the cell membrane. To confirm this finding, we extracted membrane and cytoplasmic proteins from co-expressed KCNQ1-EGFP/KCNE1-mCherry and P441L-EGFP/KCNE1-mCherry cells. A small amount of KCNQ1 protein in variant P441L expressing cells was detected in cell membranes (Figure 2H). The results indicated that the presence of KCNE1 facilitated variant P441L transport. In Co-IP experiment, compared with wild-type KCNQ1, the amount of pulled down KCNE1 with variant P441L was markedly reduced (Figures 2I,J). These findings suggested that variant P441L may affect the interaction between KCNQ1 and KCNE1. Even though KCNE1 may facilitate the transport of the variant to the membrane, KCNE1 does not completely rescue the dysfunction caused by variant P441L.

### Electrophysiological characteristics of the KCNQ1 P441L variant channel

**FIGURE 3:**
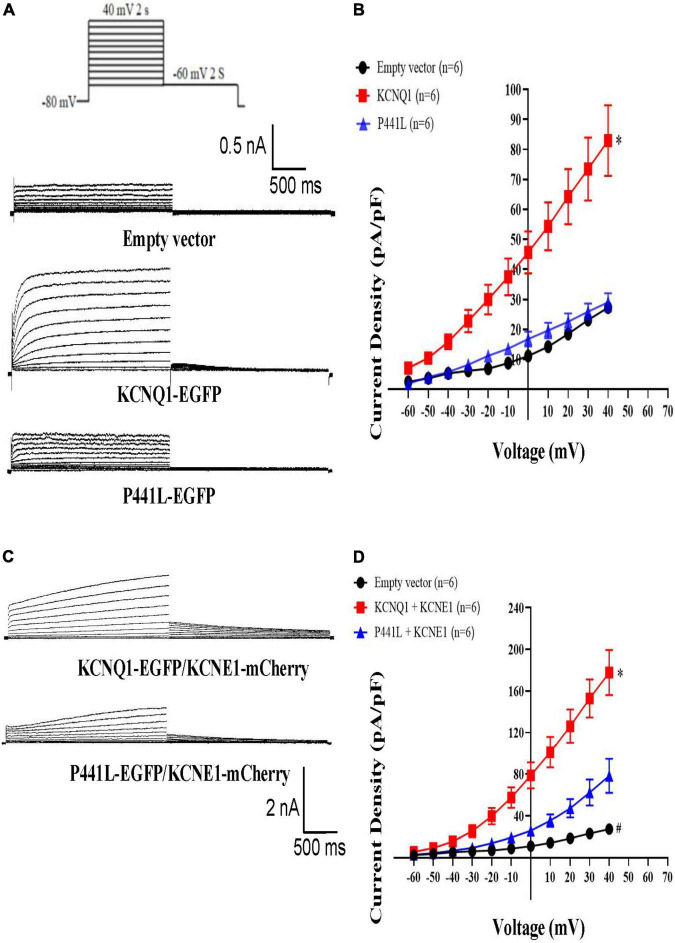
Properties of KCNQ1 and P441L mutant whole-cell currents in HEK293 cells. **(A)** The 4-s test pulse voltage protocol **(top)** and representative current traces from HEK293 cells expressing empty vectors, KCNQ1-EGFP, or P441L-EGFP. **(B)** Summary data showing whole-cell current–voltage relationships of HEK293 cells expressing empty vectors, KCNQ1-EGFP, or P441L-EGFP. Data are shown as the mean ± SEM, *n* = 6, *P < 0.05 vs. KCNQ1-EGFP. **(C)** Representative current traces from HEK293 cells co-expressing KCNQ1-EGFP/KCNE1-mCherry or P441L-EGFP/KCNE1-mCherry. **(D)** Summary data showing whole-cell current–voltage relationships of HEK293 cells expressing empty vector, KCNQ1-EGFP/KCNE1-mCherry, or P441L-EGFP/KCNE1-mCherry. Data are shown as the mean ± SEM, *n* = 6, *P < 0.05 KCNQ1-EGFP/KCNE1-mCherry vs. P441L-EGFP/KCNE1-mCherry; ^#^*P* < 0.05 Empty vector vs. P441L-EGFP/KCNE1-mCherry.

Previous studies have shown that expression of KCNQ1 with the C-terminus fused to EGFP produces currents that are not different from KCNQ1 (12, 14). We transiently transfected HEK293 cells with KCNQ1-EGFP or P441L-EGFP to form homomultimer channels. Results indicated that wild-type KCNQ1 exhibited relatively slowly activating and deactivating potassium currents in EGFP-positive cells under voltage clamp, but the cells with the P441L channel exhibited very small rapid activation currents (Figures 3A,B). When KCNQ1 and KCNE1 subunits were co-assembled, the current of the P441L-EGFP/KCNE1-mCherry channel was only half that of the KCNQ1-EGFP/KCNE1-mCherry channel (Figures 3C,D), but there is no statistical difference in channel opening speed and difficulty between the two groups (Supplementary Figure 2). This finding indicated that only a portion of the expressed P441L-EGFP/KCNE1-mCherry channels were located in the cell membrane.

## Discussion

In this study, we found a KCNQ1 missense P441L mutation at the KCNQ1 C-terminus in a 37-year-old woman diagnosed as having LQT1. The patient’s ECG showed prolong QTc intervals, and she experienced stiff limbs and convulsions with loss of consciousness. Several previous studies have shown that KCNQ1 mutations may lead to LQT1 through two mechanisms: (1) the KCNQ1 channel protein loses the ability to correctly co-assemble or to be transported to the plasma membrane; and (2) the KCNQ1 channel is unable to mediate K^+^ flow across the plasma membrane (15–17).

We demonstrated that KCNQ1 variant P441L channel protein expressed in HEK293 cells was not transported to the plasma membrane but remained in the ER. Our whole-cell patch clamp results also indicated that HEK293 cells with the KCNQ1-P441L channel expressed did not have the same K^+^ current as cells with the wild-type channel. In addition, the interaction between the KCNQ1-P441L channel protein and KCNE1 was significantly reduced. Previous studies have shown that mutations in the C-terminus of KCNQ1 are mostly in helix C and helix D (T587M, R594Q, and G619D), which may interfere with the transport of ion channel proteins to the cell membrane, resulting in a corresponding loss of function (12, 18–20). However, the mechanism by which the mutation site between helix A and helix B leads to LQT1 is unclear. The M437V mutation adjacent to the P441L mutation has been shown to affect the channel ion current mediated by KCNQ1 but it does not affect transport to the plasma membrane (21). However, we here firstly report that mutation located between helix A and helix B, markedly affected the transport of the channel subunits to the membrane and the interaction with KCNE1 and eventually led to severe LQT1.

Most of the KCNQ1 mutations associated with LQT1 are in the transmembrane segment, with only 31.2% of the pathogenic mutations in the C-terminus (4). In addition, compared with patients who have KCNQ1 mutations in the channel transmembrane region, many patients with C-terminal mutations have exhibited a lower risk of cardiac events beside T587M mutation at the C-terminus between helix C and helix D (10, 12, 22, 23). In our study, the clinical manifestations of variant P441L were severe in this patient. Genetic testing can provide valuable clinical information for long QT syndrome and provide some help in the diagnosis, treatment and prognosis of the disease, but we still have to consider the possible role of ethnicity, and the possible existence of mutations in other, untested genes should be weighed in the balance (24–26). Therefore, cardiologists stratifying risk of cardiac events should avoid discounting the pathogenic potential of C-terminal mutations in KCNQ1 because some of them, including and perhaps especially P441L, may induce a severe LQT1 phenotype with increased risk of cardiac events.

## Conclusion

In conclusion, we demonstrated for the first time that the C-terminal mutation P441L, located between helix A and helix B in the KCNQ1 protein, may lead to severe LQT1 with increased risk of cardiac events, causes trafficking defect and affects the interaction between KCNQ1 and KCNE1.

## Data availability statement

The raw data supporting the conclusions of this article will be made available by the authors, without undue reservation.

## Ethics statement

The studies involving human participants were reviewed and approved by the Medical Ethics Committee of the First Affiliated Hospital of Anhui Medical University (PJ2021-14-34). The patients/participants provided their written informed consent to participate in this study. Written informed consent was obtained from the individual(s) for the publication of any potentially identifiable images or data included in this article.

## Author contributions

HL, WD, HX, BS, and RZ contributed to the design of the study. HL, WD, HX, MD, YX, ZJ, JG, and MW performed the experiments. BS and RZ supervised the project. HL, WD, BS, and RZ wrote the manuscript. All authors participated in revising the article and approved the final manuscript.
