# Supplementary material for: Association of the P441L KCNQ1 variant with severity of long QT syndrome and risk of cardiac events
Source: Front Cardiovasc Med. 2022 Oct 31;9:922335. doi: 10.3389/fcvm.2022.922335 (PMC9659898; doi:10.3389/fcvm.2022.922335)
Supplement: Supplementary file 1 [file Data_Sheet_1.PDF]

Supplement Figure:

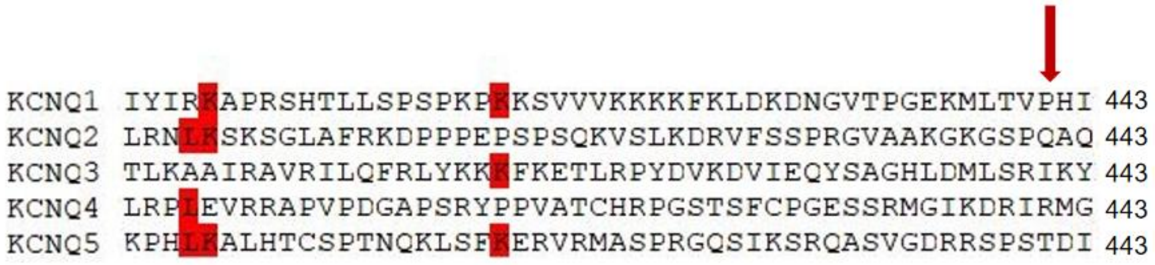

Supplement-Figure 1. Partial amino acid sequences ofhuman KCNQ family potassium channels KCNQ1–5 at the position corresponding to tyrosine 441 of human KCNQ1 (arrow). Number, the position of the last amino acid of each partial sequence. The conserved motif is outlined in black.

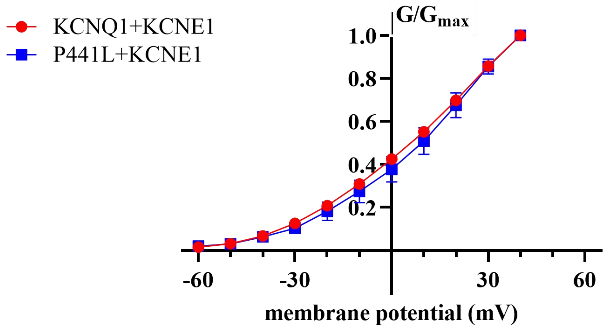

Supplement-Figure 2. Conductance－voltage relationships of KCNQ1-EGFP and P441L-EGFP LQT1 mutants co-expressed with KCNE1-mCherry in HEK293 cells (n =6). Data are shown as the mean ± SEM, n=6, *P < 0.05 vs. KCNQ1/KCNE1.
